# The rumen microbiome: an underexplored resource for novel antimicrobial discovery

**DOI:** 10.1038/s41522-017-0042-1

**Published:** 2017-12-01

**Authors:** Linda B. Oyama, Susan E. Girdwood, Alan R. Cookson, Narcis Fernandez-Fuentes, Florence Privé, Hannah E. Vallin, Toby J. Wilkinson, Peter N. Golyshin, Olga V. Golyshina, Ralf Mikut, Kai Hilpert, Jennifer Richards, Mandy Wootton, Joan E. Edwards, Marc Maresca, Josette Perrier, Fionnuala T. Lundy, Yu Luo, Mei Zhou, Matthias Hess, Hilario C. Mantovani, Christopher J. Creevey, Sharon A. Huws

**Affiliations:** 1 Institute of Biological Environmental and Rural Sciences, Aberystwyth University, Aberystwyth, Wales, SY23 3DA UK; 2 Bangor University, Bangor, Gwynedd, LL57 2DG UK; 3 Karlsruhe Institute of Technology, Hermann-von-Helmholtz-Platz 1, 76344 Eggenstein, Leopoldshafen, Germany; 4 Institute of Infection and Immunity, St. George’s University of London, Cranmer Terrace, London, SW17 0RE UK; 5 Specialist Antimicrobial Chemotherapy Unit, Public Health Wales, University Hospital of Wales, Heath Park, Cardiff, CF14 4XW UK; 6 Wageningen University & Research, 6708 WE, Wageningen, Netherlands; 7 Aix Marseille Université, CNRS, Central Marseille, iSm2, Marseille, France; 8 Centre for Experimental Medicine, School of Medicine, Dentistry and Biomedical Sciences, Queen’s University Belfast, Belfast, Northern Ireland, BT9 7BL UK; 9 School of Pharmacy, Queens’s University Belfast, Belfast, BT9 7BL, Northern Ireland, UK; 10 UC Davis, College of Agricultural and Environmental Sciences, California, 95616 USA; 11 Department of Microbiology, Universidade Federal de Viçosa, Viçosa, 36570-900 Brazil; 12 Institute for Global Food Security, School of Biological Sciences, Medical Biology Centre, Queen’s University Belfast, 97 Lisburn Road, Belfast, Northern Ireland, BT9 7BL UK

## Abstract

Antimicrobial peptides (AMPs) are promising drug candidates to target multi-drug resistant bacteria. The rumen microbiome presents an underexplored resource for the discovery of novel microbial enzymes and metabolites, including AMPs. Using functional screening and computational approaches, we identified 181 potentially novel AMPs from a rumen bacterial metagenome. Here, we show that three of the selected AMPs (Lynronne-1, Lynronne-2 and Lynronne-3) were effective against numerous bacterial pathogens, including methicillin-resistant *Staphylococcus aureus* (MRSA). No decrease in MRSA susceptibility was observed after 25 days of sub-lethal exposure to these AMPs. The AMPs bound preferentially to bacterial membrane lipids and induced membrane permeability leading to cytoplasmic leakage. Topical administration of Lynronne-1 (10% w/v) to a mouse model of MRSA wound infection elicited a significant reduction in bacterial counts, which was comparable to treatment with 2% mupirocin ointment. Our findings indicate that the rumen microbiome may provide viable alternative antimicrobials for future therapeutic application.

## Antibiotics: Potential in the rumen

Anti-microbial molecules made by microbes in the gut of ruminant animals could become new weapons against antibiotic-resistant infections. An international team of researchers led by Sharon Huws at Queen’s University Belfast, UK, identified three anti-microbial peptides in the rumen of animals such as cattle, sheep and goats. The peptides—short proteins—were highly active in laboratory trials against several clinically important drug-resistant infections. These included methicillin resistant *Staphylococcus aureus* (MRSA), a notorious cause of life-threatening infections, especially in patients with weakened immunity. There is growing interest in using peptides as alternatives to existing antibiotics. The findings, initiated by examining a ‘library’ of molecular data, suggest that the rumen is an under-explored resource that may harbor many medically useful antimicrobials. The possibilities should be investigated further, with promising molecules being tested in clinical conditions.

## Introduction

The global increase of multi-drug resistant (MDR) bacteria, combined with the decreasing number of novel and effective antibacterial agents being developed for the market, poses a serious global threat to human health.^1,2^ Urgency is required with respect to discovery and development of novel antibiotics, alongside antimicrobial stewardship and development of rapid tools to detect MDR bacteria.^3^ Antimicrobial peptides (AMPs), particularly cationic AMPs, are promising alternatives to currently available antibiotics, due to their broad spectrum activity against Gram positive and Gram negative bacteria, and their rapid mode of action on the cytoplasmic membrane, which results in a decreased rate of resistance being developed by the targeted pathogens. However, synthetic generation of AMPs has produced very few if any viable AMPs for medical use, thus there is a need to further prospect from nature.^4^

Depending on availability of resources, bacteria in communities can either be symbiotic with one another or live in competition.^5^ In order to compete, bacteria often up-regulate genes encoding AMPs to kill other bacteria in the community.^5^ The rumen is an example of such a highly competitive community with a very complex microbiome, composed of unique and diverse range of bacteria, archaea, protozoa, fungi, and viruses^6,7^ and as such is a potentially unique resource for novel AMP discovery.^8–10^ In the past few years, it has become apparent that culture dependent methods to evaluate microbial populations substantially underestimates the diversity of microorganisms present, and only around 10–20% of the species from the rumen environment have been cultured to date.^11,12^ The limitations surrounding culturability and antimicrobial discovery can be circumvented using sequence and functional-based metagenomics, which allow mining of whole microbiomes for novel bioactive compounds.^13,14^

Here, we report the discovery and characterization of three AMPs (i.e., Lynronne-1, Lynronne-2 and Lynronne-3) via functional metagenomic screening of a rumen bacterial metagenomic library and *in-silico* sequence data analysis. These novel AMPs from the rumen microbiome have the potential to treat MDR bacterial infections.

## Results

### Identification of AMPs

**Fig. 1:**
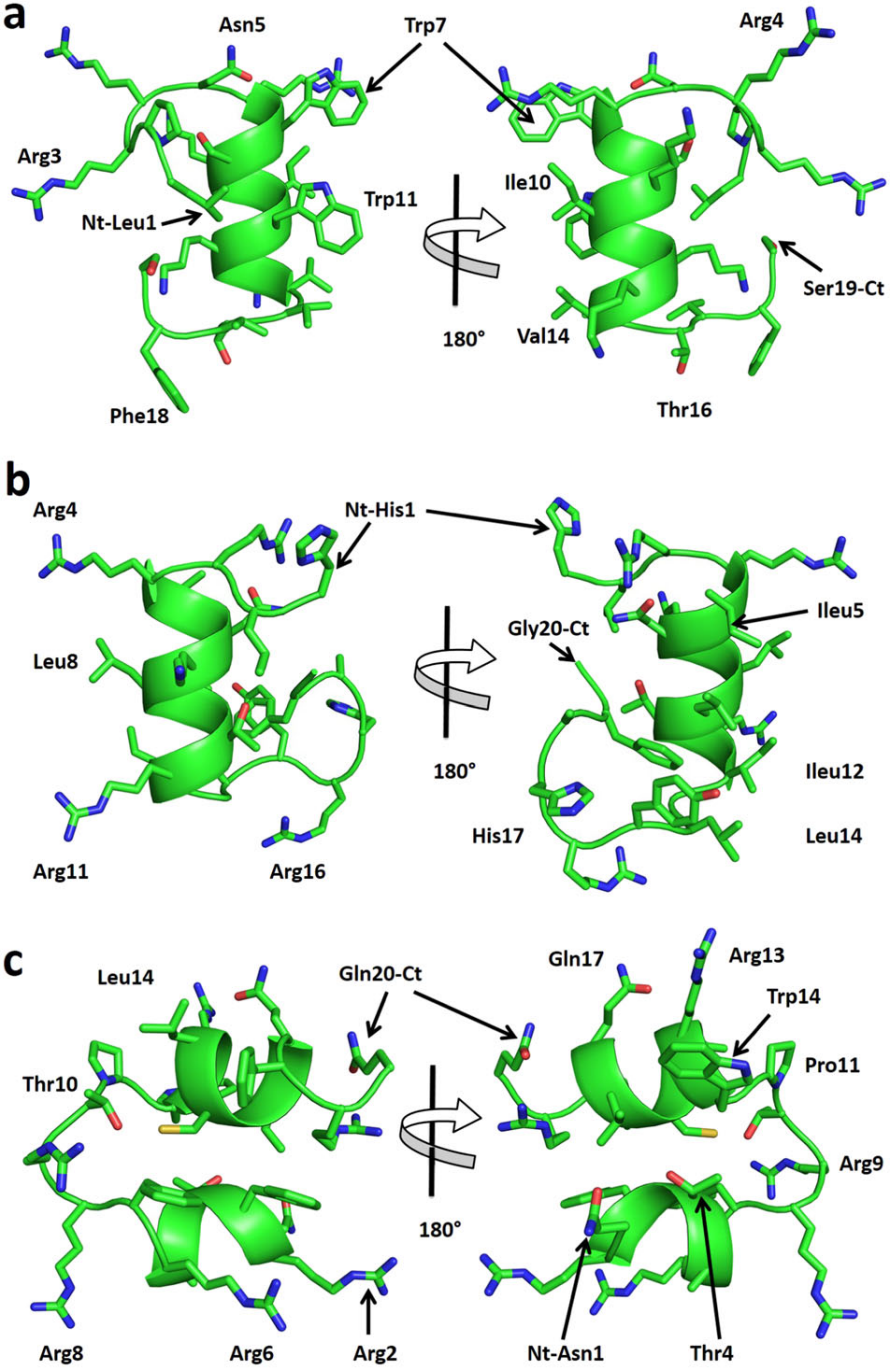
Predicted structures for peptides: Lynronne-1, Lynronne-2 and Lynronne-3. **a** Lynronne-1, **b** Lynronne-2 and **c** Lynronne-3. Main-chain and side chains depicted in ribbon and stick representation respectively and colored according to atom type: carbon, oxygen and nitrogen in green, red and blue respective. Two orientations are shown rotated about the shown axis. Ct and Nt (C and N terminals) as well as selected residues are depicted in the figure. Figures were rendered using PyMol

A metagenomic library consisting of 8448 fosmids from the plant-attached rumen microbiome^15^ was screened for antimicrobial activity by a combination of agar-based functional screening, sequencing of positive clones and bioinformatic prediction of AMP sequences. To test the antimicrobial activity of fosmid clones, lawns of pathogens including methicillin-sensitive *Staphylococcus aureus* (MSSA) RN4220, *Escherichia coli* K12, *Salmonella enterica* serovar Typhimurium SL1344, *Listeria monocytogenes* NCTC 11994 (serovar 4b) and *Enterococcus faecalis* JH2-2 were inoculated on appropriate agar medium and the clones were gently transferred on top using multichannel pin replicators. After an incubation period of 24 h at the appropriate temperatures, 255 fosmids produced clearing zones in the pathogen lawn, indicating that the metagenome clones carried inserts with antimicrobial activity. Of these clones, twenty-four were selected randomly and sequenced using the GS FLX system. A summary of the assembly metrics of the twenty-four metagenomic inserts with antimicrobial activity is provided in Supplementary Table S1. The full fosmid sequences are available in the GenBank database under the BioProject PRJNA264884; BioSample IDs: SAMN063330279 to SAMN063330302. Prediction of AMPs from the identified open reading frames (ORFs) yielded a total of 181 AMPs. A peptide library consisting of 135 of these AMPs (≤25 amino acids (AAs)) was synthesized and screened for antimicrobial activity using the SPOT technique (synthesis of large numbers of peptides on marked spots on cellulose membrane sheets allowing for subsequent highthroughput screening).^16^ Twenty-five active AMPs were identified in this screen (Supplementary Table S2). Candidates, Lynronne-1 (19 AAs: LPRRNRWSKIWKKVVTVFS-NH_2_), Lynronne-2 (20 AAs: HLRRINKLLTRIGLYRHAFG-NH_2_) and Lynronne-3 (20 AAs: NRFTARFRRTPWRLCLQFRQ-NH_2_) encoded in fosmid clones SABPL29H11, SABPL5A1 and SABPL12(2)A3, respectively and were selected for further analysis due to their broad spectrum antibacterial activity and lower minimum inhibitory concentration (MIC) values. The nucleotide and protein sequences of the ORFs from which they were derived are available in the GenBank database under accession numbers KY628802, KY628803 and KY628804, respectively. Sequences upstream and downstream of these ORFs are shown in Supplementary Fig. S1. The likely producers of Lynronne-1, Lynronne-2 and Lynronne-3 were identified as *Prevotella ruminicola* 23 (CP002006.1), Uncultured bacterium Contig939 (KC246977.1) and Uncultured bacterium Contigcl_1559 genomic sequence (KC246861.1) respectively (Supplementary Table S3 and Supplementary Fig. S2). Structural modelling using PEP-FOLD^17^ indicate that these peptides adopt a α-helical conformation of amphipathic nature, an arrangement typical of many α-helical AMPs^18^ (Fig. 1). The AMPs have a net positive charge of +6, +5 and +6, respectively with a hydrophobicity ratio of ≥40%.

### Antimicrobial activity, resistance and mechanism of action

The antibacterial activity of Lynronne-1, Lynronne-2 and Lynronne-3 against twenty-nine clinically important multidrug-resistant pathogens was explored. All three peptides had promising activity against Gram-positive pathogens, including MDR strains (Table 1). Lynronne-1 had an MIC range of 8–32, Lynronne-2: 32–256 and Lynronne-3: 32–128 of µg/ml for methicillin-resistant *S. aureus* (MRSA) strains. Since cationic peptides rarely demonstrate high in vitro antimicrobial activities seen with some conventional antibiotics, the MICs determined for Lynronne-1, Lynronne-2 and Lynronne-3 were not surprising and fall within the range of many commercially available AMPs.^19,20^

**Table 1:** Minimum Inhibitory Concentrations (MICs) against drug-resistant pathogens

Organism information			Peptides and comparator antibiotics (µg/ml)													
Lab no./Strain ID	Organism	Resistances	Cip/Lev (L)	L-1	L-2	L-3	P4	P5	P8	P10	P11	P12	P15a	P15s	Van	Mup
EMRSA-15	*S. aureus*	MRSA, Cip	>256	32	32	32	128	64	64	250	250	250		128	2	
ATCC 33591	*S. aureus*	MRSA		16	128	64									2	
USA300 BAA-1717	*S. aureus*	MRSA		8	256	64									2	0.12
Q14-0320	*S. aureus*	MRSA	64	32	>128	128	>128	>128	>128	>128	64	64	>128	>128		
Q14-0325	*S. aureus*	MRSA	0.25	32	>128	128	>128	128	128	>128	32	64	>128	64		
Q14-0352	*S. aureus*	MRSA	0.5	32	>128	128	>128	>128	128	>128	32	64	>128	>128		
Q14-0390	*S. aureus*	MRSA	64	32	>128	128	>128	128	128	>128	32	64	>128	>128		
NCTC 12493	*S. aureus*	MRSA	0.25	16	64	64	32	64	32	>128	32	16	>128	64		
ATCC 29213	*S. aureus*	Sensitive	0.25	32	>128	128	>128	128	128	>128	32	64	>128	>128		
RN4220	*S. aureus*	Sensitive	>256	32	32	32	128	64	64	256	256	256		128	1	
Q14-0095	*K. pneumoniae*	KPC	16	32	>128	32	>128	128	>128	>128	>128	>128	>128	128		
Q14-0146	*K. pneumoniae*	OXA-48	>128	32	64	64	>128	>128	>128	>128	>128	>128	>128	128		
Q14-0285	*K. pneumoniae*	VIM	32	16	64	64	64	128	128	>128	>128	>128	>128	128		
Q14-0251	*K. pneumoniae*	CTX-M	16	16	64	64	>128	>128	>128	>128	128	128	>128	128		
ATCC 700603	*K. pneumoniae*	SHV-18	0.25	16	64	32	128	>128	128	>128	128	64	>128	128		
NCTC 13442	*K. pneumoniae*	OXA-48	1	16	128	32	>128	128	>128	>128	128	>128	>128	128		
	*K. pneumoniae*	Sensitive	0.03	16	128	32	>128	>128	>128	>128	128	>128	>128	>128		
Q13-0717	*A. baumanii*	IMI, MER	16	4	8	32	>128	32	64	>128	64	64	>128	128		
Q13-0795	*A. baumanii*	IMI, MER	16	4	8	32	>128	32	64	>128	64	64	128	64		
1560739339	*A. baumanii*	OXA-23	128	4	16	32	>128	128	64	>128	128	64	128	128		
Q12-0809	*A. baumanii*	OXA-23, OXA-51	128	4	8	16	32	64	32	64	128	32	128	128		
Q12-0867	*A. baumanii*	Sensitive	0.25	4	8	8	64	16	64	128	32	16	128	64		
Q14-0208	*P. aeruginosa (CF)*	CAZ, IMI, MER	4	16	32	32	>128	128	64	>128	64	64	128	128		
Q14-0890	*P. aeruginosa (CF)*	CAZ, IMI, MER	4	32	64	16	64	64	32	128	16	32	128	64		
Q12-0535	*P. aeruginosa (CF)*	Sensitive	0.06	64	128	128	>128	128	128	125	128	32	128	128		
Q12-0581	*P. aeruginosa (CF)*	IMI, MER	2	32	128	32	128	64	128	>128	32	128	128	64		
Q12-0758	*P. aeruginosa (CF)*	CAZ, IMI, MER	2	32	64	32	128	128	128	>128	32	128	128	64		
ATCC 27853	*P. aeruginosa (CF)*	Sensitive	0.25	64	>128	128	>128	128	>128	>128	>128	128	128	128		
PA01	*P. aeruginosa (CF)*		0.5	32	128	32	512	64	32	512	512	512		128		
AMT0060	*P. aeruginosa (CF)*		0.06 (L)	32	64	128							>512	64		
C3719	*P. aeruginosa (CF)*		< 0.03 (L)	64	32	16							>512	32		
LESB58	*P. aeruginosa (CF)*		0.06 (L)	64	256	32							>512	128		
M15U114296A	*C. coli*	Cip, Tet, Ery, Amox, Gent	>128	64	>128	128	>128	128	>128	>128	>128	>128	128	128		
M15U114452A	*C. coli*	Cip, Cla, Ery	>128	64	>128	128	>128	128	>128	>128	>128	>128	128	128		
M16U109179A	*C. jejuni*	Azith, Cip, Ery, Tet	>128	64	>128	128	>128	128	>128	>128	>128	>128	128	128		
M16U109568A	*C. jejuni*	Cip	128	64	>128	64	>128	128	>128	>128	>128	>128	128	128		
NCTC 11351	*C. jejuni*			64	>128	128	>128	128	>128	>128	>128	>128	128	128		
NCTC 11161	*C. jejuni*		0.5	4	32	256										
ATCC 33292	*C. jejuni*		0.12	4	32	64										
K12	*E. coli*		0.06	32	64	64	64	64	64	256	256	256		64	128	
SL1344	*Sal. typhimurium*		0.12	32	32	64	32	64	32	64	512	256		128	256	
JH2-2	*Ent. faecalis*		64	32	32	64	64	64	64	512	256	256		128	64	
NCTC 11994	*List. monocytogenes*		64	32	32	32	64	64	64	512	256	128		128	64	

Blank: not tested

*L-1, L-2 and L-3* Lynronne-1, 2 and 3, *P4-P15a*: peptides 4–15a, *Lev (L)* Levofloxacin, *Cip* Ciprofloxacin, *Van* Vancomycin, *Mup* Mupirocin, *KPC* Klebsiella pneumoniae carbapenemase, *OXA* Oxacillin, *VIM* Metallo-β-Lactamase gene, *CTX-M* extended spectrum β-Lactamase, *SHV-18* β-Lactamase, *IMI* (imipenem), *MER* (meropenem), *CAZ* (ceftazidime), *Tet* Tetracycline, *Ery* Erythromycin, *Amox* Amoxicillin, *Gent* Gentamicin, *Azith* Azithromycin, *Cla* Clarithromycin

**Fig. 2:**
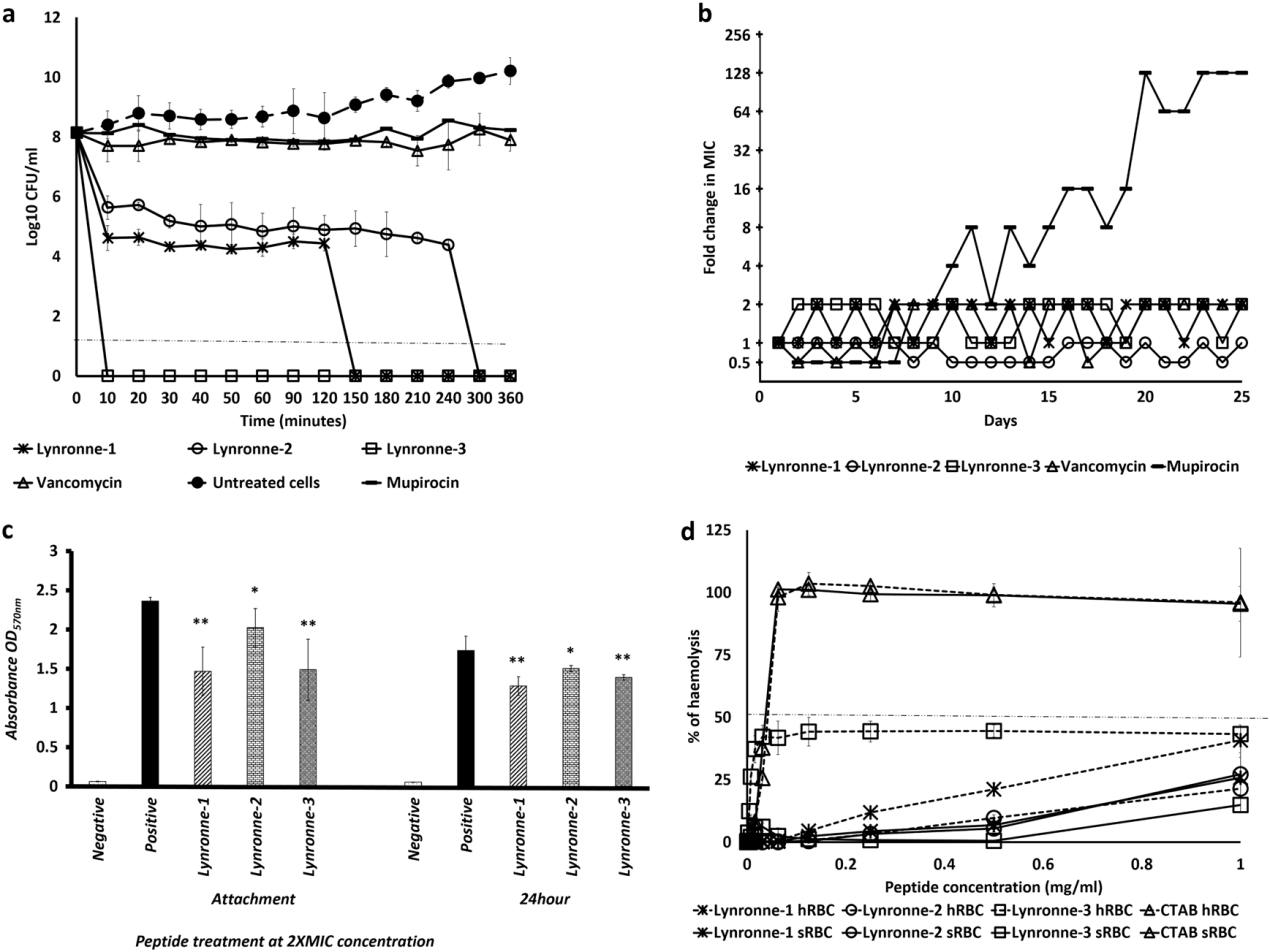
Antimicrobial activity and haemolytic activity of Lynronne-1, Lynronne-2 and Lynronne-3. **a** Time-dependent kill of MRSA USA300 by antimicrobial compounds at 3× MIC concentration. Dashed lines indicate limit of detection. **b** Resistance acquisition during serial passaging of MRSA USA300 cells in the presence of sub-MIC levels of antimicrobials. The *y* axis is the fold change in MIC during passaging. For mupirocin, 256× MIC was the highest concentration tested. The figure is representative of three independent experiments. **c** Quantification of anti-biofilm activity (MRSA 33591): * and ** *P* < 0.05 and 0.01 respectively—significantly different from untreated cells (positive). **d** Haemolytic activity, dashed line indicates 50% haemolysis. Values from at least three independent replicates; error bars represent the standard deviation)

Lynronne-1, Lynronne-2 and Lynronne-3 (at 3× MIC) had relatively faster bactericidal activity against three MRSA strains when compared to vancomycin, a first line drug for MRSA (Fig. 2a, and Supplementary Fig. S3a, b). Reductions of >8 log_10_ CFU/ml occurred within the first 10 min for Lynronne-1 and Lynronne-3, and >3 log_10_ CFU/ml in 30 min for Lynronne-2 (Fig. 2a, and Supplementary Fig. S3a, b). Vancomycin at 3× MIC produced between 1 to >4 log_10_ CFU/ml reductions depending on the MRSA strain. This can be attributed to difference in kill kinetics and mode of action.^21^ Time dependent killing of *E. coli* K12 was investigated to ascertain the efficacy of the peptides against Gram-negative bacterial strains. All three AMPs caused a rapid decrease in *E. coli* K12 viability at 3× MIC with complete cell death occurring within 10 min (see Supplementary Fig. S4a). Control agents, Polymyxin B and ciprofloxacin showed expected bactericidal activities^22^ (>4 log_10_ CFU/ml) against *E. coli* K12 at 10 min and 3 h, respectively (see Supplementary Fig. S4a).

Serial passage of MRSA strains in the presence of sub-MIC levels of Lynronne-1, Lynronne-2 and Lynronne-3 over a period of 25 days failed to produce mutants that were resistant to the AMPs (Fig. 2b and Supplementary Fig. S3c, d), suggesting a non-specific mode of action.^23^ The efficacy of Lynronne-1, Lynronne-2 and Lynronne-3 against *S. aureus* biofilm attachment and established biofilms at sub and supra-MIC concentrations were tested. MRSA ATCC 33591 strain was used in this assay as it was a better biofilm former in our hands than the other strains (results not shown). All AMPs decreased biofilm attachment at 2× MIC concentrations (Fig. 2c) and Lynronne-2 was effective even at 0.25× MIC (data not shown). Anti-biofilm activity against established biofilms was observed with all peptides at ≥2× MIC concentrations (Fig. 2c).

**Fig. 3:**
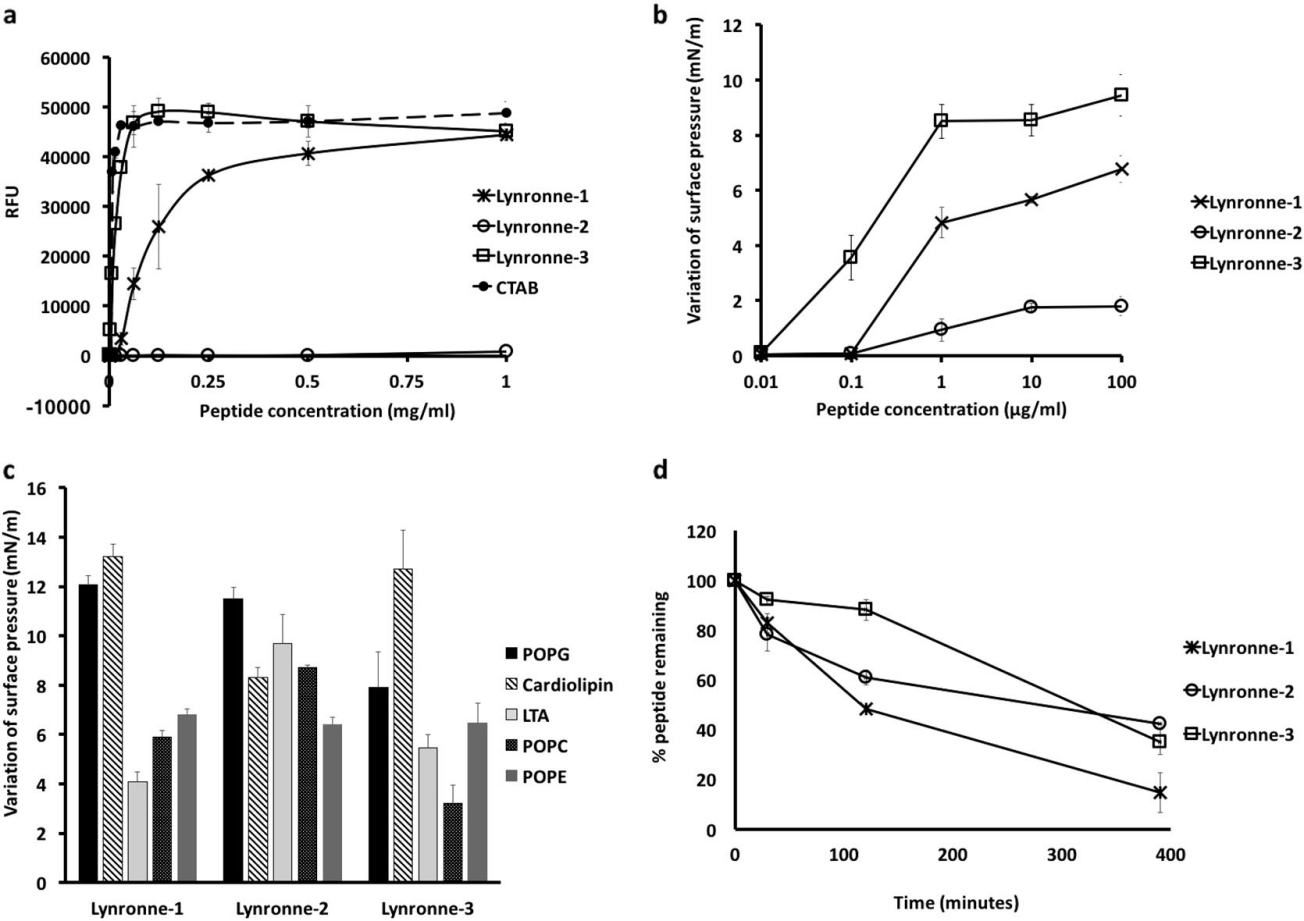
Mode of action studies of Lynronne-1, Lynronne-2 and Lynronne-3 against MRSA: **a** Membrane permeabilization activity against MRSA USA300 measured by propidium iodide assay at 10 min. **b** Dose-dependent interaction of peptides with total MRSA lipid extract, the initial surface pressure of lipid monolayer being 30 ± 0.5 mN/m. **c** Interaction of peptides (at 1 µg/ml final concentration) with pure lipids-maximal variation of surface pressure induced by the injection of peptide in lipid monolayer with initial surface pressure of 30 ± 0.5 mN/m. **d** peptide stability/degradation in 25% serum over time. Relative peptide concentrations were determined by integration of the peaks from RP-HPLC chromatograms (values from three independent replicates; error bars represent one standard deviation)

Importantly, Lynronne-1, Lynronne-2 and Lynronne-3 also showed little haemolytic activity against red blood cells (Fig. 2d). Similarly, Lynronne-1, Lynronne-2 and Lynronne-3 were less cytotoxic to mammalian HUVEC and HepG2 cells when compared to the positive control agent, Staurosporine, which had 50% growth inhibition (GI_50_), total growth inhibition (TGI) and 50% lethal concentration (LC_50_) of 0.00047, 0.0047 and 0.014 µg/ml respectively. Lynronne-2 had no cytotoxic activity against mammalian HUVEC and HepG2 cells at the highest concentration tested (128 µg/ml) while Lynronne-3 had little cytotoxicity at concentration close to the MIC with GI_50_, TGI and LC_50_ of 24.4, 61.2 and >128 µg/ml respectively. Lynronne-1 on the other hand, had low toxicity at concentrations above the MIC with GI_50,_ TGI and LC_50_ of 45.9, 67.1 and 98.1 µg/ml respectively. Whereas Lynronne-1 and 3 were able to permeabilize MRSA USA300 cell membrane, very little permeabilization activity was observed for Lynronne-2 even at >3 times its MIC concentration (Fig. 3a and Supplementary Fig. S5a), demonstrating that although antimicrobial activity of Lynronne-1 and 3 is due to pore formation, mechanism of action of Lynronne-2 may be different. In accordance with cytotoxicity results, Lynronne-1, Lynronne-2 and Lynronne-3 were less able to permeabilize eukaryotic membranes (Hep2G) compared to bacterial cell membranes (Supplementary Fig. S5b).

**Fig. 4:**
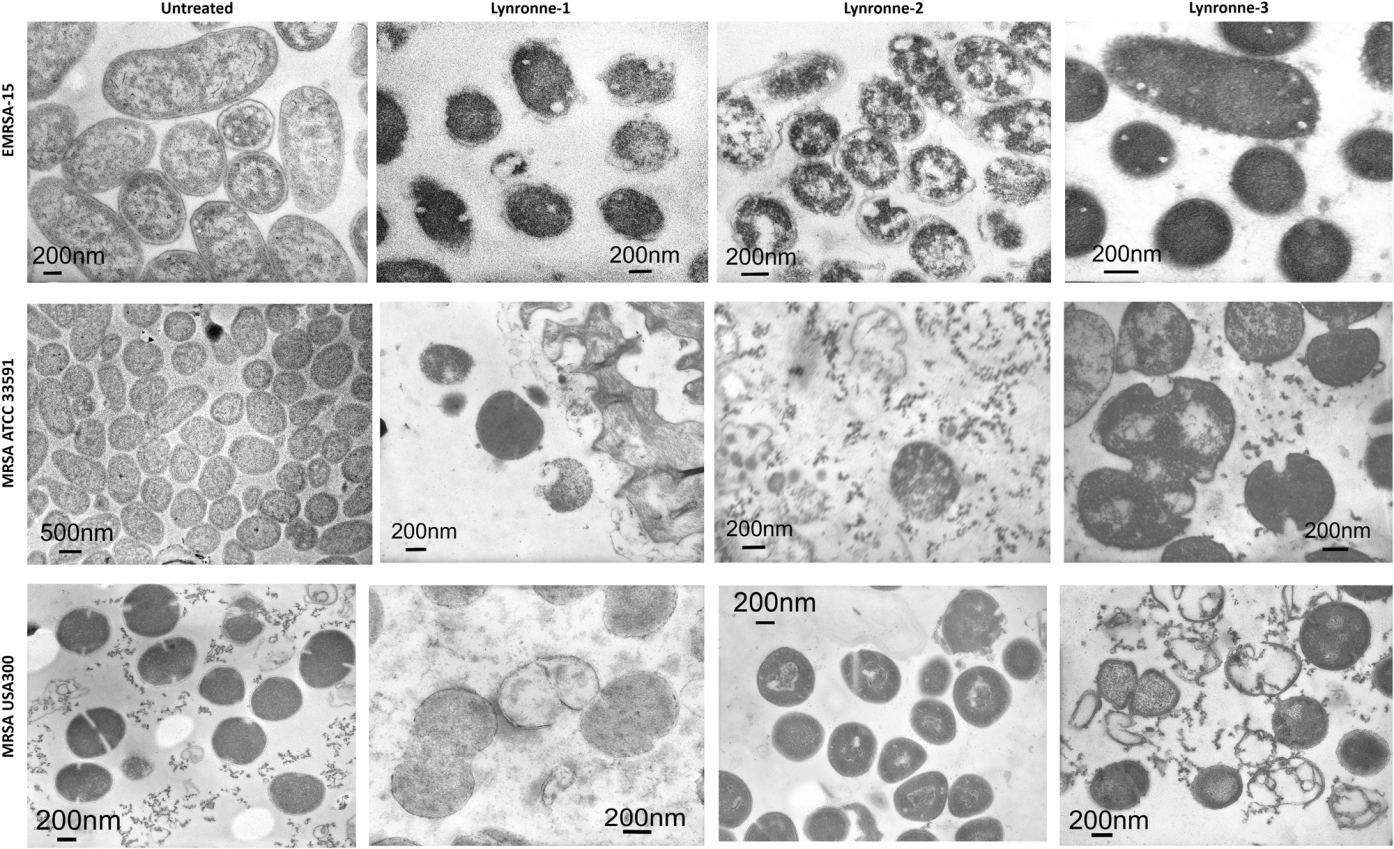
Transmission electron micrographs of MRSA cells. Representative micrographs of untreated and peptide treated MRSA cells. Cells were treated at 3× MIC peptide concentration for 1 h. Scale bars are 200 or 500 nm as shown on micrographs

Since bacterial membrane permeabilization was demonstrated by the newly identified AMPs, we used lipid monolayer (Langmuir balance) to narrow down the potential lipids that are targeted by these peptides. The use of MRSA lipid extract demonstrated a higher insertion ability of Lynronne-1 and Lynronne-3 compared to Lynronne-2 (Fig. 3b) and is in agreement with bacteria membrane permeabilization. The use of pure lipids demonstrated that Lynronne-1, Lynronne-2 and Lynronne-3 preferentially interact with lipids specific to bacteria, including 1-palmitoyl-2-oleoyl-sn-glycero-3-(phospho-rac-(1-glycerol)) (POPG), cardiolipin, lipoteichoic acid (LTA) or phosphatidylethanolamine (POPE) (also present in the inner membrane leaflet of eukaryotes) above lipids only present in eukaryotes, such as palmitoyloleoylphosphatidylcholine (POPC) (Fig. 3c and Supplementary Fig. S5c, d). This may explain the limited haemolytic and cytotoxic activity against mammalian cells in spite of their antibacterial activity. Interestingly, Lynronne-2 showed the highest affinity for LTA demonstrated by measurement of the critical pressure of insertion of the peptides in the different lipids (Supplementary Fig. S5d), suggesting that this peptide could target other molecules containing teichoic acid moieties, including cell wall teichoic acids and may also explain its antimicrobial activity in the absence of pore-formation in whole bacteria. Transmission electron micrographs of cells treated with Lynronne-1, Lynronne-2 and Lynronne-3 (at 3× MICs for 1 h) reveal varying changes in cell morphology and some cytoplasmic leakage (Fig. 4 and Supplementary Fig. S4b). MRSA USA300 cells treated with Lynronne-2 showed very little morphology changes, corresponding to the low permeabilization activity of the peptide. Further investigation into events leading up to changes in cell morphology may be required as the transmission electron microscopic analysis is semi-quantitative.

### In vivo efficacy

**Fig. 5:**
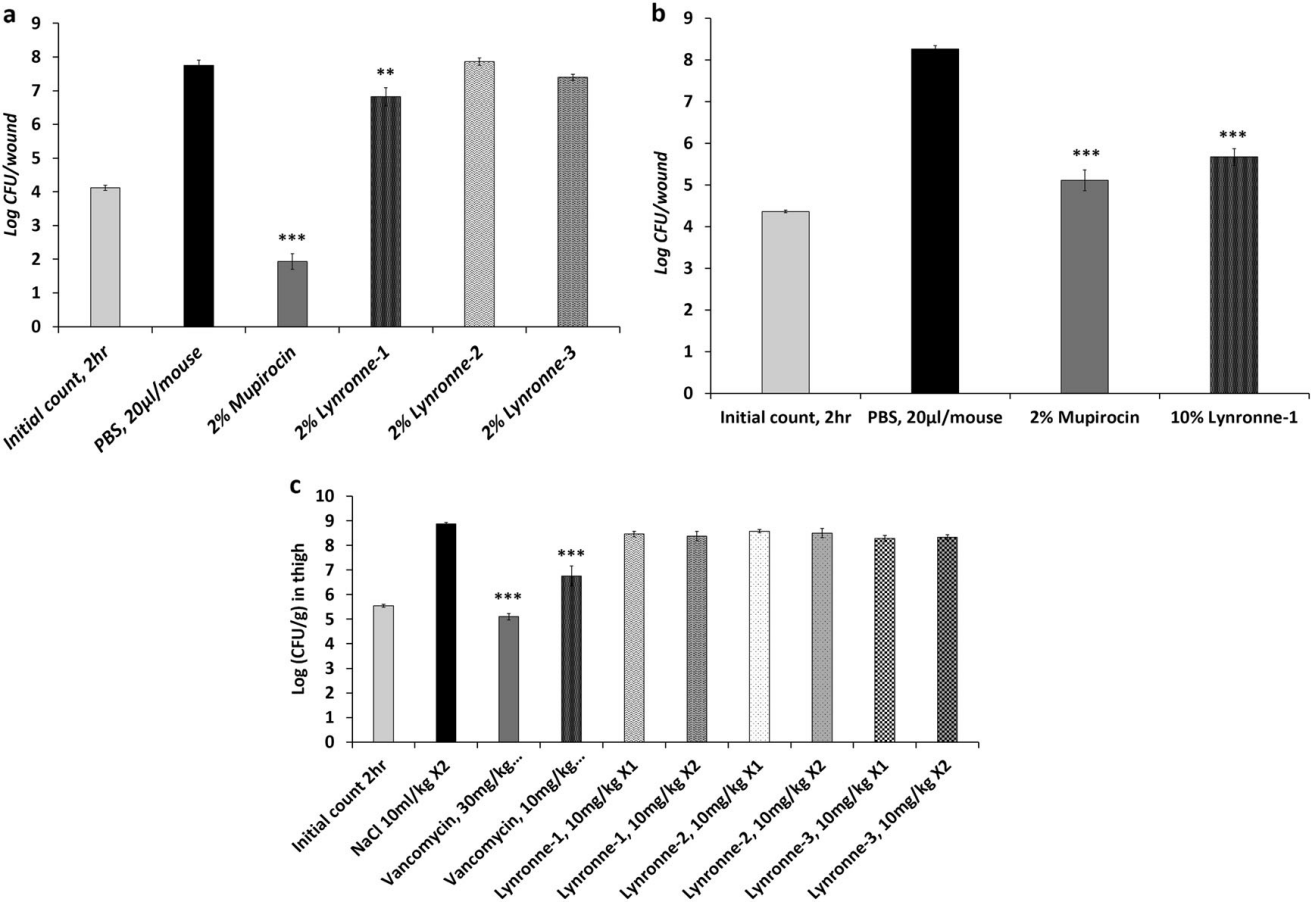
In vivo efficacy in mouse models of infection. **a** Efficacy of Lynronne-1, Lynronne-2, Lynronne-3 and mupirocin against methicillin resistant *S. aureus*, USA300 MRSA (BAA-1717), wound infection in, ICR mice. Inoculum = 1.05 × 10^5^ CFU/ml. **b** Efficacy of Lynronne-1 and mupirocin against methicillin resistant *S. aureus*, USA300 MRSA (BAA-1717). **c** Efficacy of intravenously administered Lynronne-1, Lynronne-2, Lynronne-3 and Vancomycin in the *S. aureus* (ATCC 33591) MRSA thigh infection model with neutropenic male ICR mice. Inoculum = 1.57 × 10^5^ CFU/ml, ×1 (administered once at 2 h) or ×2 (twice at 2 and 8 h post infection). *, **, *** Significant difference (*P* < 0.05, 0.01 and 0.001, respectively) compared to the control group (PBS or 0.9% NaCl) was determined by one-way ANOVA followed by the use of a Dunnett’s test

Given the promising in vitro activity of Lynronne-1, Lynronne-2 and Lynronne-3, we investigated their in vivo therapeutic potential within a murine model of MRSA skin infection.^24^ Topical administrations of Lynronne-1 (2% w/v), in a group of mice with MRSA infected wounds (five mice/group) once daily for 3 days was associated with a significant decrease (*P* < 0.01) in the colony count relative to the control group treated with phosphate-buffered saline (PBS, pH 7.4) (Fig. 5a). However, this decrease was less than a 2-log reduction (≥99%) in colony counts. No significant decreases in colony counts were observed in the animal groups treated with Lynronne-2 (2% w/v) and Lynronne-3 (2% w/v). The reference, 2% mupirocin ointment (Dermasafe^®^ Ointment) was associated with a significant reduction (>2-log reduction, and *P* < 0.001) in bacterial counts, consistent with historical data.^24^ Given this promising in vivo result for Lynronne-1, we further tested its efficacy at a higher dose (10% w/v) in the wound model. Lynronne-1 at 10% (w/v) was associated with a significant (99%) reduction in bacterial density (≥2-log reduction, and *P* < 0.001) and comparable to mupirocin (2%) treatment (Fig. 5b). These findings suggest that Lynronne-1 can effectively reduce the bacterial load in MRSA infected wounds.

The efficacy of Lynronne-1, Lynronne-2 and Lynronne-3 were subsequently tested in a murine model of MRSA deep thigh infection.^25^ The maximum tolerable dose of all three AMPs was predetermined to be 10 mg/kg in vivo in mice. Test animals were inoculated intramuscularly (IM) in the right thigh with 0.1 ml/thigh of *S. aureus* MRSA ATCC 33591 (inoculum size of 1.57 × 10^5^ CFU/mouse) followed by treatment with Lynronne-1, Lynronne-2 and Lynronne-3 and the comparator antibiotic, vancomycin. Intravenous (IV) administrations of Lynronne-1, Lynronne-2 and Lynronne-3 at 10 mg/kg once or twice (2 h or 2 and 8 h post infection) were not associated with any significant antimicrobial effects compared to the vehicle/control group (0.9% NaCl). Vancomycin administered twice at 30 mg/kg IV) elicited a significant reduction (≥99%, 2-log reduction in CFU/g and *P* < 0.001). However, vancomycin at 10 mg/kg IV (administered twice) only elicited a significant reduction (*P* < 0.001) but not a 2-log reduction in colony counts compared to the control group (Fig. 5c).

Upon testing for stability of peptides, we observed that Lynronne-1 rapidly degraded in the presence of serum with only 48 and 15% of the parent peptide remaining after 2 h and 6 h respectively. Lynronne-2 and Lynronne-3 were relatively more stable with ≥60 and 42%, as well as ≥88 and 34% after 2 h and 6 h respectively (Fig. 3d). A similar pattern was observed when these peptides were treated with trypsin (Supplementary Fig. S6), a common feature among peptides that are susceptible to degradation by peptidases.^26^ This susceptibility to degradation may explain the poor efficacy of the peptides when administered intravenously.

## Discussion

AMPs are promising candidates for overcoming the critical and growing challenge of bacterial resistance to currently utilized antibiotics. The high cost and low success rate associated with the identification of novel and affective AMPs from natural sources has hindered the search for novel AMPs in the past. This hurdle has been overcome by new technological advances and improvements in traditional recombinant methods.^23^ Using a combination of functional metagenomics and computational approaches, we were able to show that the rumen microbiome is a promising resource for bio-prospecting novel AMPs, and these three peptides (Lynronne-1, Lynronne-2 and Lynronne-3), identified in this study are potential therapeutic candidates.

Lynronne-1, Lynronne-2 and Lynronne-3 are efficacious against clinically important drug-resistant pathogens in in vitro models of infection. In addition, Lynronne-1 decreased bacterial counts in MRSA wound infections using a murine model, similar to commercially used mupirocin ointment, suggesting that it could be used topically in the treatment of MDR bacterial infections. Lynronne 1, 2 and 3 displayed low haemolytic activity against blood cells and negligible cytotoxicity against mammalian cells. Results presented here suggest that loss of cell viability after exposure to Lynronne-1, Lynronne-2 and Lynronne-3 among many factors is due to membrane permeabilization, which contributes to membrane disruption and leakage of cell content. In addition to their broad antibacterial spectrum, selectivity and rapid killing of bacterial cells, Lynronne-1, Lynronne-2 and Lynronne-3 also showed a low tendency to select for resistance in the bacteria strains tested. The antimicrobial compounds discovered here are novel and demonstrate potent activity against clinically relevant human pathogens, rendering them as potential therapeutics. The identification of these novel AMPs support the hypothesis that the rumen is a promising resource for the discovery of novel antimicrobials with clinical relevance.

## Methods

### Functional antimicrobial activity screening of a rumen metagenomic library

A metagenomic library consisting of 8448 clones from a plant-associated rumen microbiome was prepared previously.^15^ The clones were transferred gently using multichannel pin replicators (Molecular Devices Ltd, Berkshire UK) onto Luria–Bertani agar plates containing a lawn (500 µl) of *S. aureus* (MSSA) RN4220, *E. coli* K12, *Salmonella enterica* serovar Typhimurium SL1344, *E. faecalis* JH2-2 and *L. monocytogenes* NCTC 11994 serovar 4b (OD_600_ = 1) using sterile pin replicators. Plates containing these pathogens were incubated at 37 °C (or 30 °C for *L. monocytogenes*) for 24 h, and clearing zones around the fosmid clones were considered as indicative of clones containing gene inserts coding for antimicrobial compounds. Assays were performed in sextuplicates to ensure reproducibility.

### Sequence-based screening of putative antimicrobial positive fosmid clones

Fosmids with putative antimicrobial activity were sequenced using the Genome Sequencer FLX platform (454 Life Sciences) according to manufacturer guidelines. Following sequence assembly using the default parameters on the gsAssembler software v.2.5.3 (Roche Life Sciences), sequences were trimmed and vector sequence were removed using BioEdit (version 7.1.11).^27^ VecScreen on NCBI was used to search the sequences for vector contamination. ORFs were determined using NCBI’s ORF finder on the NCBI blast suite (v2.2.28).^28^

### In silico identification of AMPs

Prediction of AMP sequences within the identified ORFs was achieved using a variety of software available at various public databases. Specifically, we used the antimicrobial peptide database,^29^ the Antimicrobial Sequence Scanning System (AMPA),^30^ BACTIBASE,^31^ collection of anti-microbial peptides (CAMP),^18,32^ and antibacterial peptides (AntiBP).^33^ Only ORFs that were predicted to encode putative AMPs within at least two of the utilized databases were explored further. The active sites within positive ORFs were predicted using AMPA^30^ and make up the peptides included in the peptide library to be screened for antimicrobial activity.

### Peptide synthesis and antimicrobial screening

The peptides in the created peptide library were synthesized based on the SPOT technique and pure peptides (≥95% purity) were synthesized on resin using solid phase Fmoc peptide chemistry.^16^ The 135 peptides in the peptide library  were screened for antimicrobial activity using both luminescent (*Pseudomonas aeruginosa* strain H1001)^34^ and non-luminescent (epidemic methicillin resistant *S. aureus* EMRSA-15, *Sal. typhimurium* and *E. coli* K12) bacterial strains. Activity against non-luminescent bacteria strains was assessed by fluorescence readings (excitation/emission spectra of 560/590 nm) after the addition of resazurin dye (100 µm final concentration). The results from the screen were analysed using the MATLAB extension Gait-CAD as previously described.^35^ Concentration values with a 75% reduction of luminescence or fluorescence (IC75) in relation to untreated controls were computed. Three peptide activity classes: active (IC75 ≤ 0.25), semi-active (0.25 < IC75 ≤ 0.50) and inactive IC75 > 0.5 were assigned against all microorganisms.

### Determination of MIC

MICs were determined using a modified broth microdilution method^36^ in cation adjusted Mueller Hinton broth (MHB) following the International Organization for Standardization 20776-1 standard for MIC testing with a final bacterial inoculum concentration of 5 × 10^5^ CFU/ml.^37^ Peptides dissolved in sterile distilled water and comparator antibiotics dissolved in the appropriate solvents^38^ were added to sterile U-bottom polypropylene 96-well microplates at desired concentrations. MIC was defined as the lowest concentration of peptide or antibiotic, which inhibited the visible growth of bacteria after 18–24 h incubation at appropriate temperatures.

### Time kill kinetics

Assessment of the bactericidal activity of peptides and comparator antibiotics was performed as previously described^39^ using exponential-phase cultures of pathogens grown in MHB (1 × 10^8–10^ CFU/ml). Peptides were added at concentrations 3 times their MIC values. Experiments were performed in quadruplicates, and CFU/ml was calculated at different time points after over overnight incubation.

### Selection for AMP resistance

To evaluate if populations of AMP-resistant bacteria could be selected, cultures were continuously exposed to Lynronne-1, Lynronne-2 and Lynronne-3 for a duration of 25 days, as previously described.^40^ Briefly, broth microdilution susceptibility testing was performed using a standard doubling-dilution series of Lynronne-1, Lynronne-2 and Lynronne-3 concentrations on day 1. Following incubation of the cultures for 24 h, and determination of the MIC, the well that contained the highest concentration of AMPs permitting growth was diluted 1: 1000 in MHB and used to provide the inoculum for the next MIC assay; this process was repeated daily for 25 days.

### Erythrocyte leakage assay

The haemolytic activity of peptides was determined by measuring haemoglobin leakage from human and sheep erythrocytes (Divbioscience, NL) after 1 h incubation at OD_450_nm. Cetyltrimethylammonium bromide (CTAB), a non-specific detergent was also included as a positive control. Triton-X 100 (0.1% v/v) was also used as an indicator of 100% haemolysis while PBS treatment represented auto-haemolysis.

### Cytotoxicity of peptides

Cytotoxic activity of the identified peptides was determined using HUVEC and HEPG2 cells as described previously.^41,42^ Three dose response parameters 50% growth inhibition (GI_50_), total growth inhibition (TGI) and 50% lethal concentration (LC_50_) were calculated for each experimental agent and cellular tumor assay results are presented as the percent cell growth or percent tube length of specific activity. Significant responses were defined when observed cell growth was ≤50% and tube length was ≤70% using the proliferation and tube formation assays respectively. Permeabilization of eukaryotic membrane by Lynronne-1, Lynronne-2 and Lynronne-3 was also investigated using HepG2 cells as described previously.^43^

### Membrane permeabilization and peptide-lipid interactions

Membrane permeabilsation was evaluated using propidium iodide assay as previously explained,^43^ with CTAB as positive control. Peptide–lipid interaction was measured using reconstituted lipid monolayer.^44^ Total lipid extract from overnight liquid cultures of MRSA USA300 was obtained by Folch extraction, resuspended in chloroform and stored at −20 °C under nitrogen conditions. Pure bacterial and eukaryotic lipids, POPG, cardiolipin, LTA, POPE and POPC (Avanti Polar Lipid USA) were reconstituted in chloroform at 1 mg/ml and stored at −20 °C under nitrogen. Using a 50 µl Hamilton’s syringe, total MRSA lipid extract or pure lipids were spread at the surface of PBS creating a lipid monolayer at the air-water interface until an initial surface pressure of 30 ± 0.5 mN/m was reached. This corresponds to a lipid packing density theoretically equivalent to that of the outer leaflet of the cell membrane.^45^ After 5–10 min of incubation allowing evaporation of the solvent and stabilization of the initial surface pressure, peptides were injected into the PBS (pH 7.4, volume 800 µl) sub-phase using a 10 µl Hamilton syringe. The variation of the surface pressure caused by peptide injection was then continuously monitored using a fully automated microtensiometer (µTROUGH SX, Kibron Inc., Helsinki, Finland) until reaching equilibrium (maximal surface pressure increase usually obtained after 15–25 min). Critical pressure of insertion of each peptide in the different lipids was also determined by changing the initial pressure of lipid monolayer (from 10 and 30 mN/m) and measuring the variation of pressure caused by the injection of peptide (at 1 µg/ml final concentration). All experiments were carried out in a controlled atmosphere at 20 °C ± 1 °C and data were analyzed using the Filmware 2.5 program (Kibron Inc., Helsinki, Finland). Variation of surface pressure was plotted as a function of initial surface pressure and critical pressure of insertion was calculated as the theoretical value of initial pressure of lipid monolayer not permissive to peptide insertion, i.e., a variation of pressure equal to 0 mN/m. The accuracy of the system under our experimental conditions was determined to be ± 0.25 mNm/1 for surface pressure measurements.

### Serum stability and trypsin degradation assays

The stability of Lynronne-1, Lynronne-2 and Lynronne-3 in the presence of serum was investigated by monitoring the concentrations of the remaining soluble peptides by reverse phase high-performance liquid chromatography (RP-HPLC), as described previously by Nguyen and colleagues.^26^ Degradation of Lynronne-1, Lynronne-2 and Lynronne-3 in the presence of trypsin was adapted from a previously described method.^46^ Briefly, trypsin (5 µl of 0.5 ng/ml) and 37.5 µl trypsin activation buffer (50 mM Tris, 2 mM CaCl_2_, pH 7.8-8) was added to 7.5 µl of 5 mg/ml Lynronne-1, Lynronne-2 and Lynronne-3 and incubated at 37 °C for different time points (0, 1, 3, and 24 h). An aliquot from each reaction mixture at 0 h was prepared to allow matrix-assisted laser desorption/ionization time-of-flight (MALDI-TOF) mass spectrometric verification of the synthetic peptides prior to incubation. Trypsin action buffer (5 µl) and peptides without trypsin was used as negative control. After the various incubation times, the reaction mixtures were acidified by addition of an equal volume (50 µl) of 10% (v/v) trifluroacetic acid (TFA) to stop further enzyme activity. Lynronne-1, Lynronne-2 and Lynronne-3 degradation products were evaporated to dryness and reconstituted in acetonitrile/water/TFA (40/59.5/0.5%, v/v/v). Samples (1 µl) were carefully placed onto a stainless steel (MALDI) target, covered with 1 µl of matrix (53 mM a-cyano-4-hydroxycinnamic acid in acetonitrile/water/TFA, 70/29.97/0.03%, v/v/v) solution before analysis by mass spectrometry.

### Molecular modelling of AMP 3D structures

The modelling of the 3D conformation of peptides was carried out using the de novo structural prediction method PEP-FOLD.^17^ The best 3D models for each peptide was selected according to PEP-FOLD score, based on the OPEP force field.^47^ Results were visualized using the PyMOL v1.7.6 program.^48^

### Transmission electron microscopy

Effects of AMPs on bacterial cell morphology was investigated using transmission electron microscopy as described by Huws et al.^49^ Bacterial cultures in mid-log phase were treated with Lynronne-1, Lynronne-2 and Lynronne-3 (at 3× MIC for 1 h) and then fixed with 2.5% (v/v) glutaraldehyde. Cells were post-fixed with 1% osmium tetroxide (w/v), stained with 2% (w/v) uranyl acetate and Reynold’s lead citrate and observed using a JEOL JEM1010 transmission electron microscope (JEOL Ltd, Tokyo, Japan) at 80 kV.

### Quantification of anti-biofilm activity

The effectiveness of Lynronne-1, Lynronne-2, and Lynronne-3 to prevent (a) biofilm attachment and (b) disrupt established biofilms was measured using a 96-well biofilm model.^50^ Briefly, MRSA cultures grown in brain heart infusion (BHI) broth were washed, resuspended to an OD_600nm_ = 0.02 in BHI broth and (a) treated with peptides (at 1/4×, 1/2×, 1×, 2× and 4× MIC) and incubated at 37 °C for 24 h or (b) incubated at 37 °C for 24 h to allow establishment of biofilms, washed in PBS to remove planktonic cells, and further incubated with peptides (at 1/4×, 1/2×, 1×, 2× and 4× MIC) in fresh BHI broth at 37 °C for 24 h. The biofilms were washed in PBS to remove planktonic cells, fixed with methanol, stained with 0.5% (w/v) crystal violet and re-solubilized with 33% (v/v) acetic acid. The optical density of biofilms was measured at 570 nm.

### In vivo efficacy of peptides in mouse models of MRSA skin and thigh infections

The efficacy of Lynronne-1, Lynronne-2 and Lynronne-3 for treating a wound infection caused by USA300 community-associated methicillin resistant *S. aureus* (MRSA, strain BAA-1717) was evaluated using a murine wound model with ICR mice.^24^ A sharp punch (ID 12 mm) was used to create excisional skin wounds in groups of five female ICR mice (5–6 weeks old). The mice were inoculated topically on the wound area (5 µl per wound) with 1.05 × 10^5^ CFU/mouse of strain BAA-1717 suspended in 5 µl PBS. Peptides (2 or 10% w/v) in PBS (pH 7.4), control (PBS), and mupirocin 2% (Dermasafe^®^ ointment) were topically applied (20 µl/mouse) directly to wounds 2 h post infection and once daily thereafter for three consecutive days. Test animals were sacrificed and the wound tissue was excised for enumeration of bacteria cells (CFU/wound). A two-log reduction in the bacterial count (≥99% reduction) relative to the control group indicates significant activity. Statistical significance (*P* < 0.05) was performed with one-way analysis of variance (ANOVA) followed by use of the Dunnett’s method.

In the thigh infection model, using neutropenic mice,^25^ groups of five male ICR mice weighing 22 ± 2 g were inoculated intramuscularly (IM) with *S. aureus* MRSA, ATCC 33591 (1.57 × 10^5^ CFU/mouse (0.1 ml/thigh)) in the right thigh. Lynronne-1, Lynronne-2 and Lynronne-3 at 10 mg/kg were intravenously (IV) administered once 2 h post infection or twice 2 and 8 h post-infection. The reference substance, vancomycin, was also intravenously injected 2 and 8 h post-infection at 10 and 30 mg/kg. Test animals were sacrificed 26 h after inoculation and the infected thighs were excised and the bacterial burden (CFU/gram) was determined.

### Data availability

The datasets generated and/or analysed during the current study are available under the study name (SRP099633 Cow rumen Metagenome) in the GenBank database, (https://www.ncbi.nlm.nih.gov/).

## Electronic supplementary material

Supplementary Material
