# Supplementary material for: The rumen microbiome: an underexplored resource for novel antimicrobial discovery
Source: NPJ Biofilms Microbiomes. 2017 Dec 1;3:33. doi: 10.1038/s41522-017-0042-1 (PMC5711939; doi:10.1038/s41522-017-0042-1)
Supplement: Supplementary file 1 — Supplementary Material [file 41522_2017_42_MOESM1_ESM.doc]

## Supplementary Material

**Supplementary Table S1: Assembly metrics of selected clones after shotgun sequencing using the Roche GS FLX system**

| **Fosmid clone**  **ID** | **GenBank**  **BioSample ID** | **Total number of reads** | **Total number of base pairs** | **Number of contigs** | **Total length of contigs (bp)** | **Insert size from chosen contig (bp)** | **GC content of insert (%)** | **Number of ORFs in contig** |
| --- | --- | --- | --- | --- | --- | --- | --- | --- |
| SAB PL4 N2 | SAMN063330279 | 26413 | 17318197 | 1102 | 2102927 | 25841 | 56.2 | 132 |
| SAB PL5 A1 | SAMN063330280 | 47599 | 30986482 | 691 | 4325733 | 67719 | 52.7 | 328 |
| SAB PL5 A2 | SAMN063330281 | 22427 | 14871695 | 1218 | 2450352 | 38145 | 51.5 | 194 |
| SAB PL5 C17 | SAMN063330282 | 5733 | 3699135 | 76 | 131887 | 12070 | 52.1 | 53 |
| SAB PL12(1) H18 | SAMN063330283 | 10168 | 6616004 | 203 | 263016 | 401 | 43.8 | 6 |
| SAB PL8 L14 | SAMN063330284 | 42326 | 26689339 | 1258 | 3662870 | 8036 | 47.9 | 45 |
| SAB PL9 A12 | SAMN063330285 | 4707 | 3113068 | 85 | 90709 | 2240 | 53.6 | 11 |
| SAB PL9 B3 | SAMN063330286 | 15899 | 10345946 | 387 | 572057 | 31665 | 48.2 | 160 |
| SAB PL9 F16 | SAMN063330287 | 10035 | 6672761 | 302 | 448066 | 13987 | 51.9 | 84 |
| SAB PL12(1) C3 | SAMN063330288 | 6626 | 4307213 | 82 | 121460 | 27728 | 52.6 | 149 |
| SAB PL12(1) D18 | SAMN063330289 | 10742 | 6936903 | 284 | 392503 | 8036 | 47.9 | 44 |
| SAB PL12(2) A3 | SAMN063330290 | 32909 | 21294400 | 1261 | 3447744 | 27125 | 47.7 | 168 |
| SAB PL12(2) G13 | SAMN063330291 | 17172 | 10597944 | 885 | 1449480 | 16816 | 51.1 | 34 |
| SAB PL12(2) J19 | SAMN063330292 | 26621 | 16046320 | 1290 | 2782997 | 31708 | 49.4 | 39 |
| SAB PL19 A3 | SAMN063330293 | 18860 | 11251168 | 645 | 992753 | 5795 | 53.4 | 33 |
| SAB PL27 G15 | SAMN063330294 | 22181 | 13408286 | 890 | 1518463 | 29772 | 45.5 | 61 |
| SAB PL27 G18 | SAMN063330295 | 14752 | 9075890 | 552 | 817667 | 24482 | 39.5 | 99 |
| SAB PL27 H14 | SAMN063330296 | 20027 | 12073019 | 950 | 1581893 | 12018 | 50.2 | 68 |
| SAB PL27 L10 | SAMN063330297 | 14117 | 8650852 | 349 | 514455 | 36694 | 51.0 | 193 |
| SAB PL27 L13 | SAMN063330298 | 14336 | 8770311 | 353 | 544607 | 35970 | 52.6 | 182 |
| SAB PL27 L17 | SAMN063330299 | 17428 | 10680537 | 751 | 1223750 | 25007 | 47.5 | 124 |
| SAB PL29 H11 | SAMN063330300 | 16143 | 10004756 | 648 | 988569 | 33998 | 49.3 | 176 |
| SAB PL29 I11 | SAMN063330301 | 21218 | 12829662 | 1101 | 1996994 | 23851 | 43.6 | 118 |
| SAB PL29 M21 | SAMN063330302 | 13816 | 8488354 | 581 | 842746 | 1609 | 56.6 | 7 |

**Supplementary Table S2: All active peptides in peptide library screen**- All 25 peptides in peptide library with ≥75% reduction of luminescence/fluorescence (IC75) in relation to untreated controls. Three activity classes: active (IC75≤0.25), semi-active (0.25<IC75≤0.50) and inactive IC75>0.5.

| **Peptide ID** | **Number in peptide library** | **Sequence** | **Length (AA)** | **IC75 values indicating activity classes** | | | |
| --- | --- | --- | --- | --- | --- | --- | --- |
|  |  |  |  | ***E. coli*** | **EMRSA-15** | ***Sal. typhimurium*** | ***P. aeruginosa* H1001** |
| Lynronne-1 | 124 | LPRRNRWSKIWKKVVTVFS | 19 | 0.06 | 0.38 | 0.24 | 0.14 |
| Lynronne-2 | 58 | HLRRINKLLTRIGLYRHAFG | 20 | 0.09 | 0.29 | 0.09 | 0.14 |
| Lynronne-3 | 91 | NRFTARFRRTPWRLCLQFRQ | 20 | 0.16 | 0.65 | 0.41 | 0.54 |
| P4 | 110 | VLHTGYRKFLHRSKRFFHLR | 20 | 0.09 | 0.32 | 0.27 | 0.43 |
| P5 | 100 | TMSLRFWRWKVR | 12 | 0.11 | 0.55 | 0.44 | 0.62 |
| P8 | 108 | AWRWKAFRNCWRVRSSSL | 18 | 0.17 | 0.78 | 3.81 | 0.40 |
| P10 | 48 | RSITRPVLVRRRWRVRPVF | 19 | 0.20 | 0.68 | 0.48 | 0.61 |
| P11 | 135 | SIKILKIYFIQGKRHWSF | 18 | 303.61 | 11.53 | 17.09 | 0.25 |
| P12 | 22 | QVRWWGRYWRRKWATCR | 17 | 0.31 | 0.33 | 0.46 | 0.31 |
| P15a | 15 | GTAWRWHYRARS | 12 | 0.34 | 0.89 | 0.67 | 0.78 |
| P15s | 96 | KFVRLKIYCRDKNKGRGISF | 20 | 0.64 | 5.34 | 7.79 | 0.43 |
| P1 | 69 | THRLRRWCRARGLAR | 15 | 0.05 | 1.03 | 1.04 | 0.71 |
| P6 | 111 | LTKKTKKQKRNLVGTT | 16 | 0.12 | 1.15 | 0.36 | 0.39 |
| P9 | 86 | LIRCSRTCLQYKTSRFMRW | 19 | 0.19 | 1.15 | 0.79 | 0.58 |
| P11 | 128 | RICRTRLTRRAGNSL | 15 | 0.22 | 0.93 | 0.63 | 1.69 |
| P14 | 80 | MRILSIIRWTRMKKSSA | 17 | 0.62 | 0.44 | 0.82 | 0.31 |
| P16 | 72 | VGVKRRLKCLLSLRS | 15 | 0.34 | 7.65 | 1.02 | 0.45 |
| P17 | 47 | RRLRTTTKLPPV | 12 | 0.35 | 1.99 | 0.73 | 1.24 |
| P18 | 87 | TTAPCKCWIGLRRCFK | 16 | 0.36 | 3.77 | 1.99 | 1.55 |
| P19 | 70 | RLLLVMIGLRSKIKWHSGI | 19 | 0.72 | 0.96 | 10000000000.00 | 0.39 |
| P20 | 133 | THILLLRLRKKVMS | 14 | 0.63 | 0.40 | 0.70 | 0.67 |
| P21 | 109 | SRATWARVRRLGLYG | 15 | 0.41 | 0.84 | 0.69 | 0.85 |
| P23 | 68 | AVWMTRSCVIWKR | 13 | 0.69 | 2.48 | 6.98 | 0.47 |
| P24 | 105 | LLMRKLIKGYGYLFGKGKRKKR | 22 | 0.62 | 0.71 | 1.30 | 0.47 |
| P25 | 76 | MAKLLRLDKKRNKFLCFV | 18 | 1.02 | 11.03 | 4.91 | 0.49 |

**Supplementary Table S3: BLASTN hits showing the most similar homologs for all selected clones contigs:** The likely producers of identified genes. (Accessed July 2013).

| **Plate/clone ID** | **Description** | **Max score** | **Total score** | **Query cover** | **E-value** | **Identity** | **Accession** |
| --- | --- | --- | --- | --- | --- | --- | --- |
| SAB PL4 N2 | *Prevotella ruminicola* 23, complete genome | 2542 | 7478 | 31% | 0 | 92% | CP002006.1 |
| SAB PL5 A1 | Uncultured bacterium Contig939 genomic sequence | 6739 | 6739 | 5% | 0 | 100% | KC246977.1 |
| SAB PL5 A2 | *Prevotella ruminicola* 23, complete genome | 1581 | 9168 | 32% | 0 | 82% | CP002006.1 |
| SAB PL5 C17 | *Acinetobacter baumannii* AB0057, complete genome | 1819 | 1819 | 7% | 0 | 99% | CP001182.1 |
| SAB PL12(1) H18 | *Prevotella ruminicola* 23, complete genome | 160 | 160 | 35% | 2.00E-35 | 86% | CP002006.1 |
| SAB PL8 L14 | *Acinetobacter baumannii* AB0057, complete genome | 1833 | 1833 | 11% | 0 | 99% | CP001182.1 |
| SAB PL9 A2 | *Morganella morganii* subsp. *morganii* KT, complete genome | 3538 | 23606 | 100% | 0 | 94% | CP004345.1 |
| SAB PL9 B3 | *Prevotella ruminicola* 23, complete genome | 1127 | 1791 | 6% | 0 | 81% | CP002006.1 |
| SAB PL9 F16 | *Prevotella ruminicola* 23, complete genome | 469 | 670 | 9% | 4.00E-127 | 77% | CP002006.1 |
| SAB PL12(1) C3 | Uncultured organism XynA gene, complete cds | 544 | 544 | 3% | 2.00E-149 | 76% | JX154664.2 |
| SAB PL12(1) D18 | *Acinetobacter baumannii* AB0057, complete genome | 1833 | 1833 | 11% | 0 | 99% | CP001182.1 |
| SAB PL12(2) A3 | Uncultured bacterium Contigcl_1559 genomic sequence | 14515 | 14588 | 24% | 0 | 99% | KC246861.1 |
| SAB PL12(2) G13 | Uncultured bacterium Contigcl_138 genomic sequence | 21309 | 21309 | 66% | 0 | 99% | KC246851.1 |
| SAB PL12(2) J19 | Uncultured bacterium Contigcl_138 genomic sequence | 24211 | 24286 | 39% | 0 | 99% | KC246851.1 |
| SAB PL19 A3 | *Prevotella ruminicola* 23, complete genome | 2250 | 2250 | 33% | 0 | 87% | CP002006.1 |
| SAB PL27 G15 | *Prevotella ruminicola* 23, complete genome | 6334 | 20112 | 74% | 0 | 87% | CP002006.1 |
| SAB PL27 G18 | *Edwardsiella ictaluri* 93-146, complete genome | 137 | 137 | 0% | 1.00E-26 | 81% | CP001600.2 |
| SAB PL27 H14 | Uncultured bacterium Contig1335 genomic sequence | 9642 | 9642 | 41% | 0 | 99% | KC247026.1 |
| SAB PL27 L10 | *Prevotella ruminicola* 23, complete genome | 2532 | 6713 | 21% | 0 | 80% | CP002006.1 |
| SAB PL27 L13 | *Prevotella ruminicola* 23, complete genome | 6753 | 28065 | 82% | 0 | 82% | CP002006.1 |
| SAB PL27 L17 | *Prevotella ruminicola* 23, complete genome | 5614 | 10809 | 45% | 0 | 81% | CP002006.1 |
| SAB PL29 H11 | *Prevotella ruminicola* 23, complete genome | 1377 | 4261 | 12% | 0 | 89% | CP002006.1 |
| SAB PL29 I11 | *Coprococcus* sp. ART55/1 draft genome | 548 | 737 | 5% | 1.00E-150 | 75% | FP929039.1 |
| SAB PL29 M21 | *Prevotella ruminicola* 23, complete genome | 931 | 931 | 99% | 0 | 77% | CP002006.1 |

**
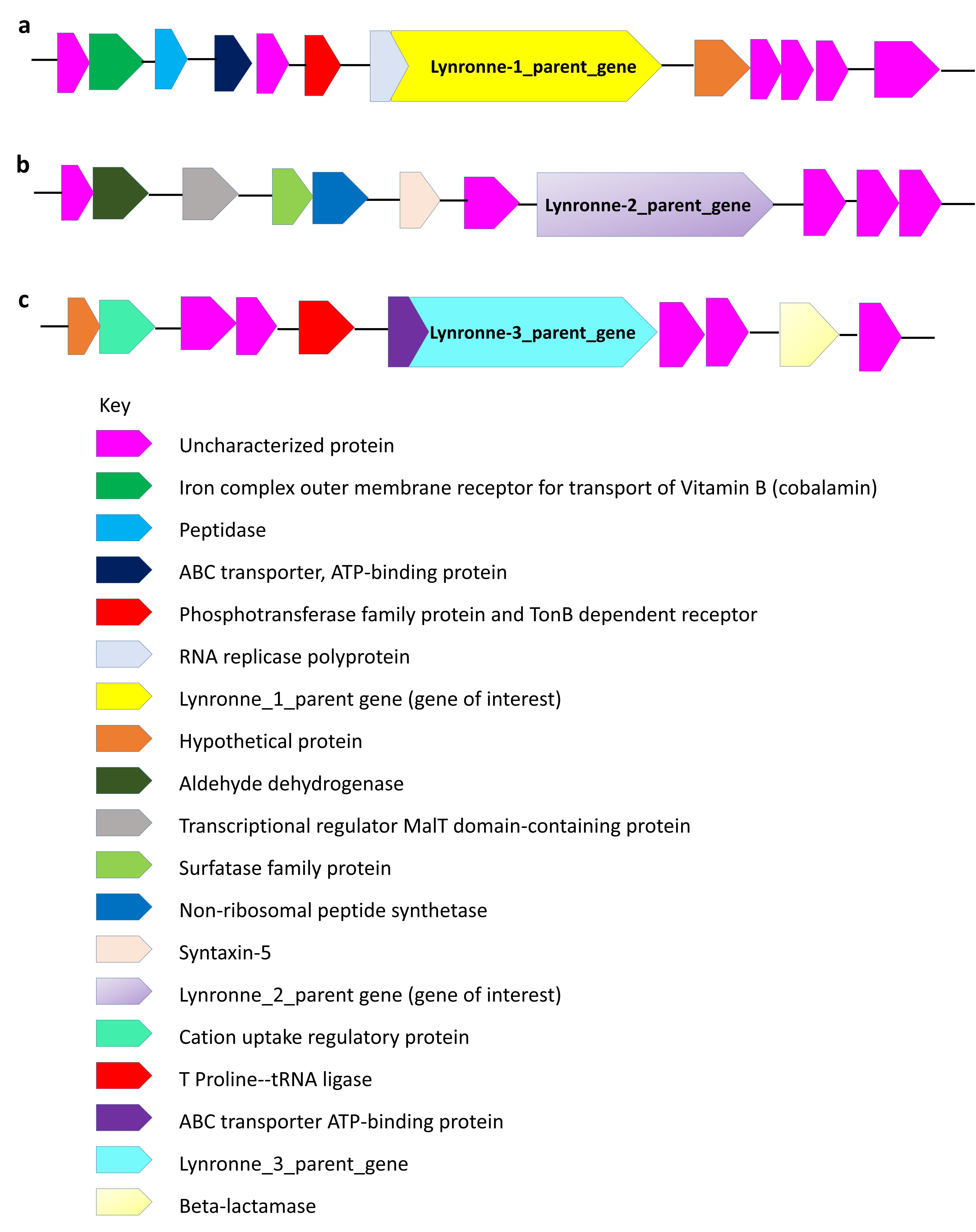
**

**Supplementary Figure S1: Sequences upstream and downstream of parent gene for a)** Lynronne-1, **b)** Lynronne-2 and **c)** Lynronne-3 predicted by NCBI blastp searches.


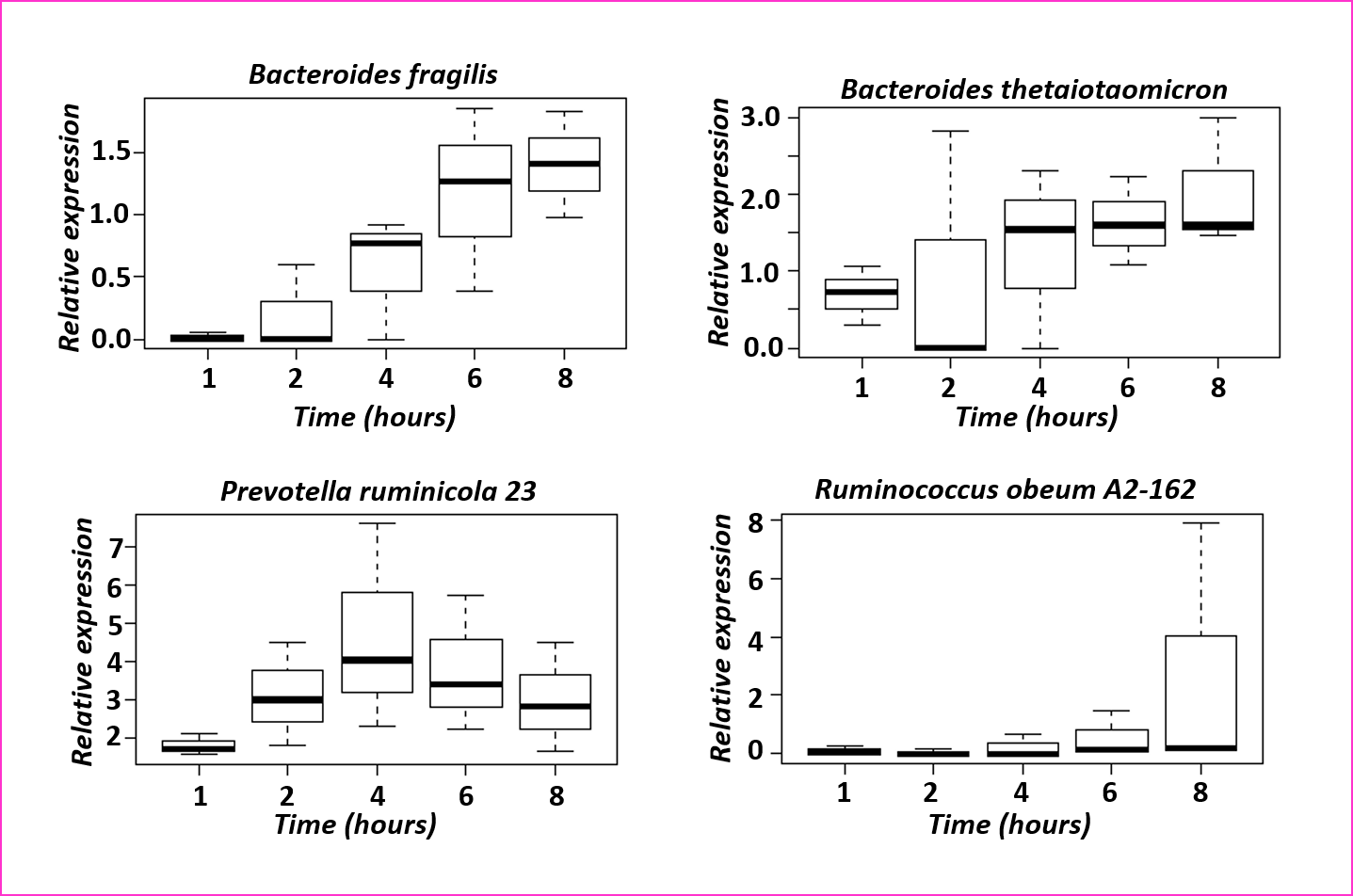


**Supplementary Figure S2: Changes in Lynronne-1 (ORF/parent gene) expression profile within perennial ryegrass attached microbiome over time.**

Based on gene expression/abundance in certain ruminal species. The x-axis represents the five hourly sample point 1, 2, 4, 6 and 8h. The y-axis represents the relative expression based on the amount of fragments aligned by the assembler during the sequencing process. Three cows were considered in this experiment and thus, three different sums of expression values are generated for each hourly sample point. These three sums form the boxplots. The different homologies and abundance in the expression of Lynronne-1 ORF from which was derived in different genera of rumen bacteria may be an indication that posttranslational modifications may occur in these genes.

**
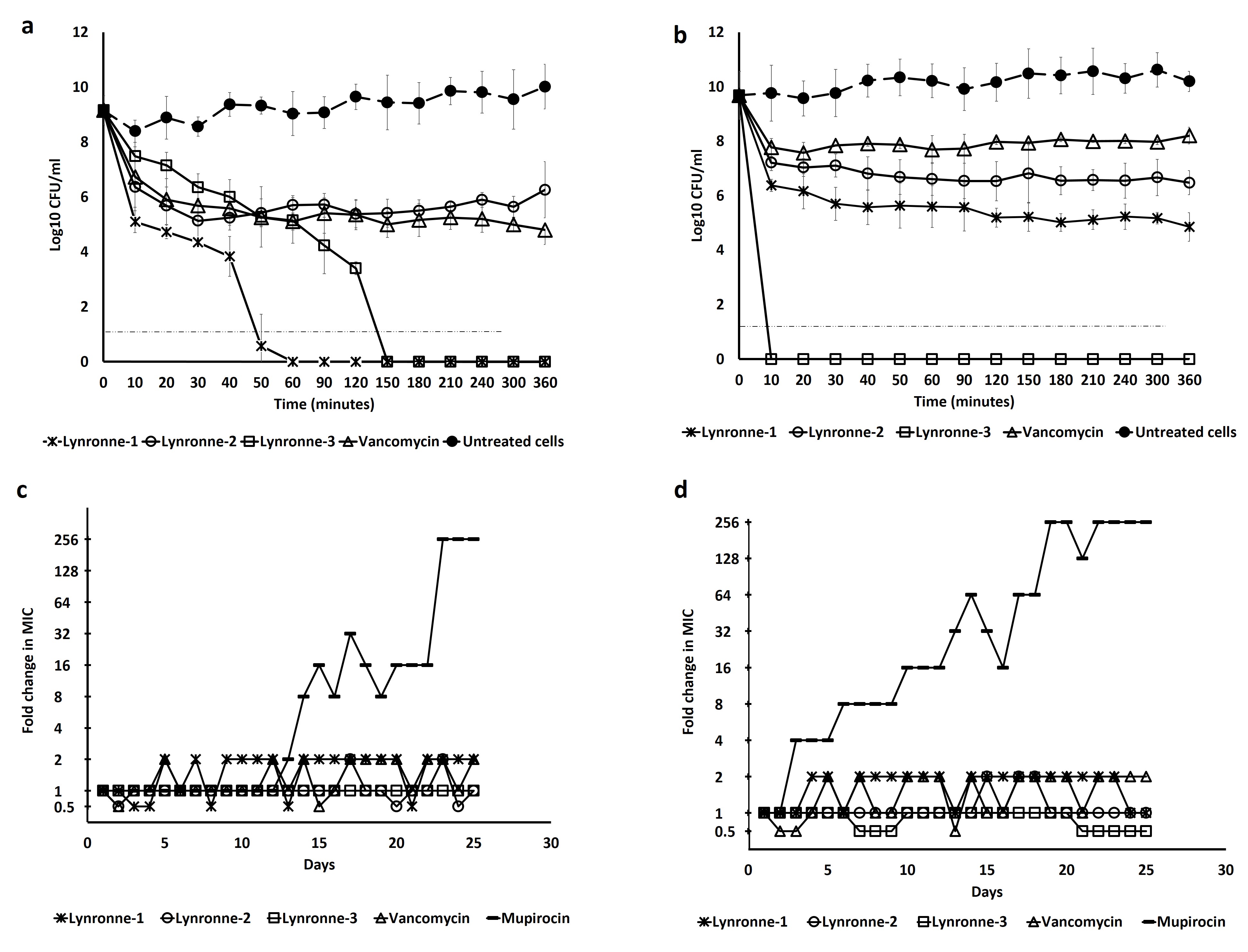
**

**Supplementary Figure S3: Antimicrobial activity of peptides: a)** Time dependent kill of EMRSA-15 **b)** Time dependent kill of MRSA ATCC 33591 by antimicrobial compounds at 3x MIC concentration. Dashed lines indicate limit of detection. Values from three independent replicates; error bars represent one standard deviation. Resistance acquisition during serial passaging of **c)** EMRSA-15 and **d)** MRSA ATCC 33591 cells in the presence of sub-MIC levels of antimicrobials. The y axis is the fold change in MIC during passaging. For mupirocin, 256x MIC was the highest concentration tested. The figure is representative of 3 independent experiments**.**


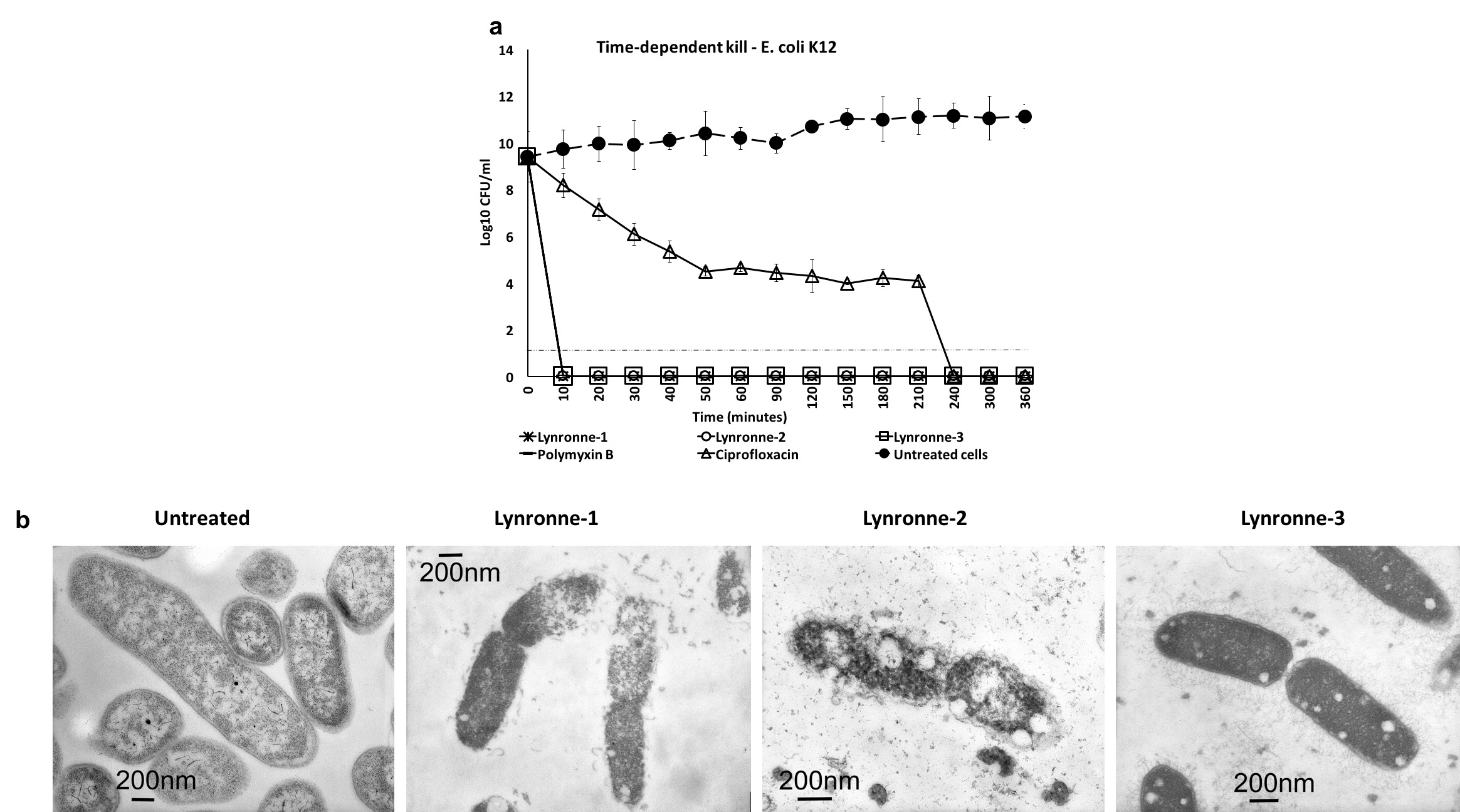


**Supplementary Figure S4: a)** Time dependent kill of *E. coli* K12 by antimicrobial compounds (values from four independent replicates; error bars represent the standard deviation), dashed line indicates limit of detection **b)** Representative TEM images of *E. coli* K12 following 60-minute exposure to peptides at 3x MIC concentrations. Scale bars are 200 nm as shown on micrographs.

**
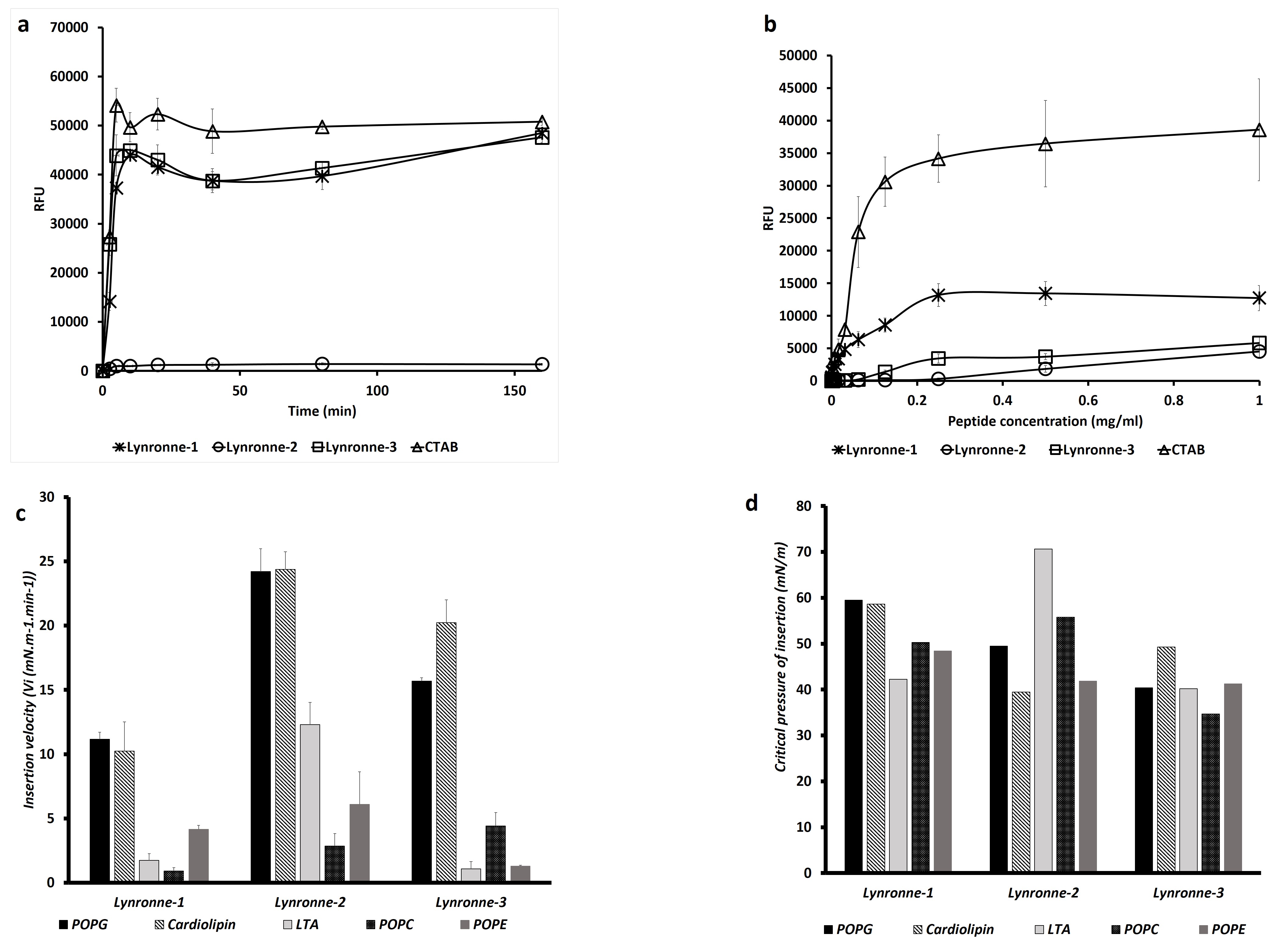
**

**Supplementary Fig S5. Mode of action of peptides: a)** Time dependent membrane permeabilzation of MRSA USA 300 cells by peptides at 1 mg/ml. **b)** Dose-effect of peptides on HepG2 permeabilization measured by propidium iodide assay at 10 mins. **c)** Interaction of peptides (at 1 *µ*g/mL final concentration) with pure lipids- Insertion velocity into pure lipids and **d)** Critical pressure of insertion (mN/m) of Lynronne-1, 2 and 3 obtained by measuring the variation of surface pressure induced by the injection of peptide in lipid monolayer with various initial surface pressures.

**
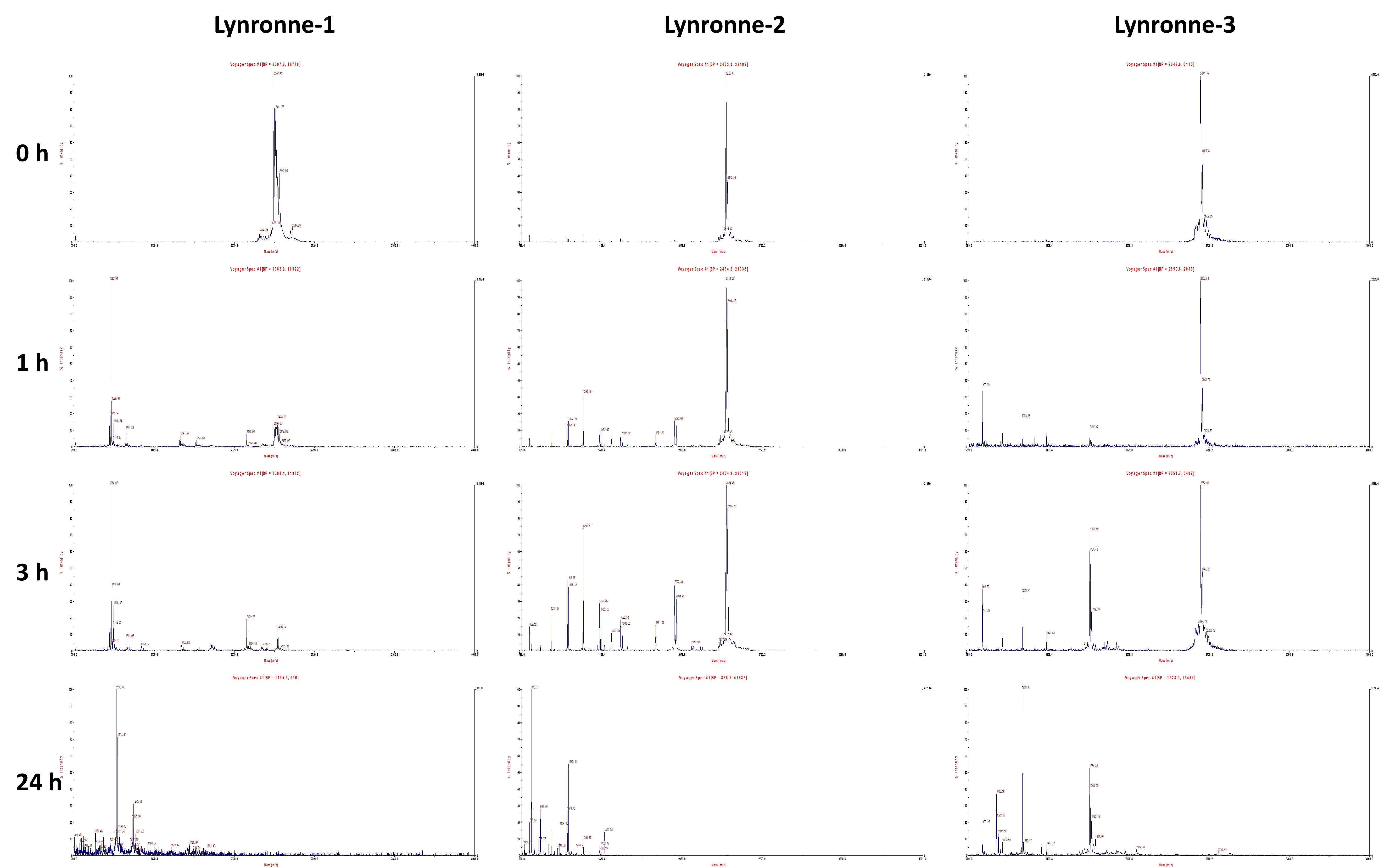
**

**Supplementary Figure S6: Peptide stability/degradation in the presence of trypsin.** An example set of MALDI-TOF chromatograms from various time points of peptides incubated with trypsin (final concentration of 0.05 ng/ml) at 37°C, showing the non-degradation/degradation of the intact peptide and appearance of its partially digested products.
